# Giant Right Atrial Aneurysm Accompanying Intrahepatic Cholestasis

**DOI:** 10.1155/2018/9025907

**Published:** 2018-02-28

**Authors:** Ayse Sulu, Osman Baspinar, Selim Kervancıoglu, Samil Hizli

**Affiliations:** ^1^Department of Pediatric Cardiology, Gaziantep University Medical Faculty, Gaziantep, Turkey; ^2^Department of Radiology, Gaziantep University Medical Faculty, Gaziantep, Turkey; ^3^Department of Pediatric Gastroenterology, Kecioren Training and Research Hospital, Ankara, Turkey

## Abstract

Right atrial aneurysms were first described in 1955, and subsequently, only a few cases have been reported. The etiology of this condition is unknown. Its comorbidity with cholestasis has not previously been reported in the literature. An 11-month-old baby female, who was an offspring from a first-cousin marriage, was referred to our hospital for investigation of jaundice. She underwent echocardiography because of a heart murmur, and this revealed a giant right atrial aneurysm. In addition, her liver biopsy confirmed the diagnosis of progressive familial intrahepatic cholestasis (PFIC) type 3. Although both conditions are rare, we found their comorbidity interesting and are thus reporting the case.

## 1. Introduction

Giant right atrial aneurysms were first described in 1955 by Bailey [1], and very few cases have subsequently been reported. Previous case reports range from those of *in utero* diagnosis in fetuses to diagnosis in adults. While the etiology is unknown, intrinsic structural protein defects and abnormal collagen structure are thought to be the causative factors [2]. Histopathologic reports define collagen plaques, smooth muscle cells, and atrial myocytes with intervals. Half of the patients with this condition are asymptomatic. The remainder, who are symptomatic, may experience atrial arrhythmias, palpitations, shortness of breath, or chest pain. The diagnosis is based on echocardiography and may be confirmed with noninvasive imaging techniques such as 3-dimensional computed tomography (CT) angiography and cardiac magnetic resonance imaging (MRI). Cardiac catheterization is not always necessary. Some cases were diagnosed following surgery because of Ebstein's anomaly. The differential diagnosis includes Ebstein's anomaly. Possible complications of the condition include arrhythmias, thrombosis, and rupture [3, 4].

Progressive familial intrahepatic cholestasis is a rare type of cholestasis. It has an autosomal recessive pattern of inheritance. There are three types of progressive familial intrahepatic cholestasis: type 1 and type 2 have an early onset starting in the neonatal period, while type 3 has a late onset. The fibrosis in these patients is progressive and eventually develops into liver insufficiency requiring liver transplantation [5].

## 2. Case Report

**Figure 1:**
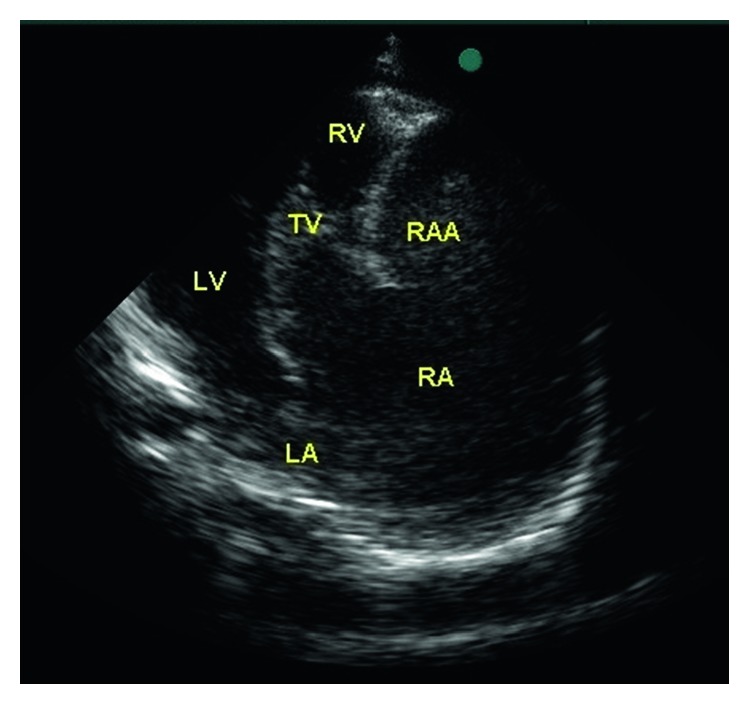
Transthoracic echocardiography of giant right atrial aneurysm associated with the right atrium. LA: left atrium, LV: left ventricle, RA: right atrium, RAA: right atrial aneurysm, RV: right ventricle, TV: tricuspid valve.

**Figure 2:**
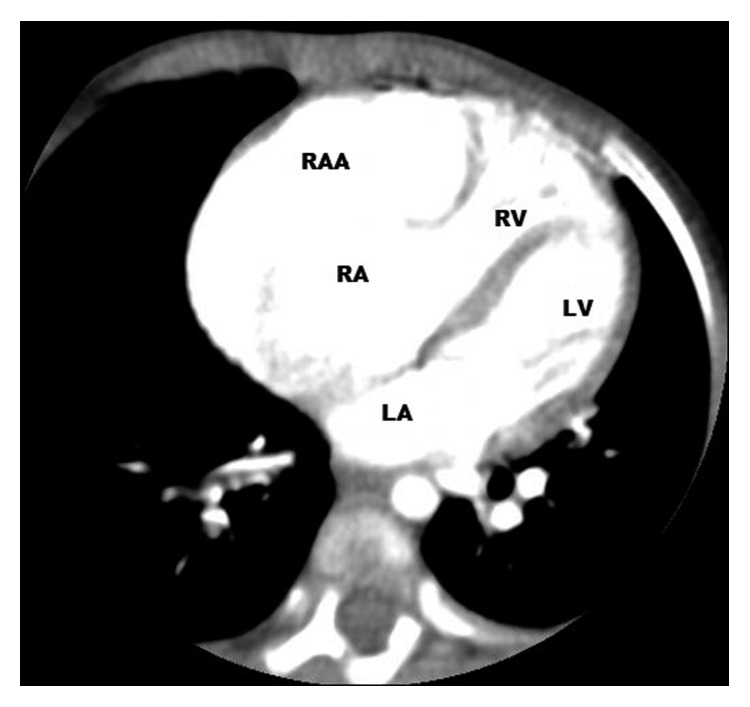
Right atrial aneurysm by CT angiography. LA: left atrium; LV: left ventricle; RA: right atrium; RAA: right atrial aneurysm; RV: right ventricle.

**Figure 3:**
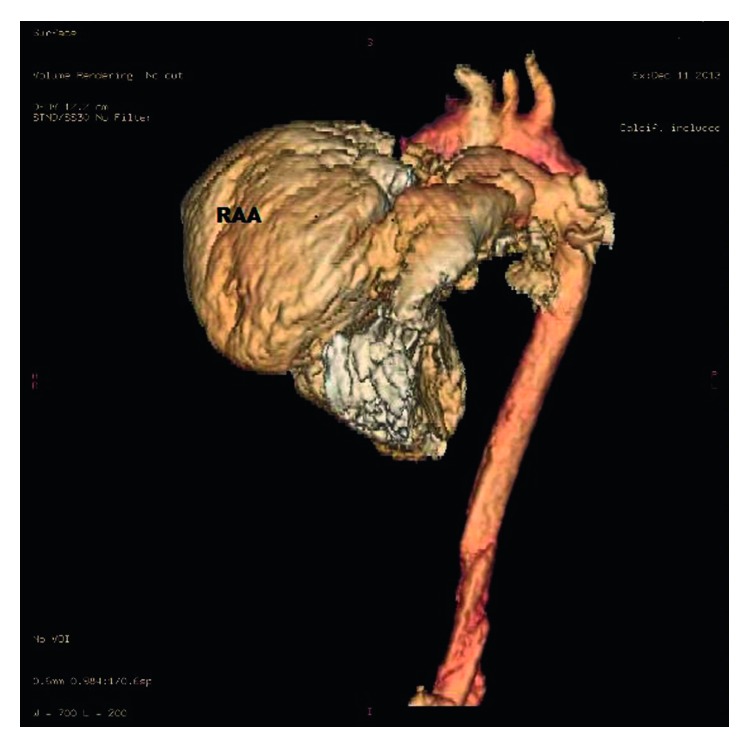
Right atrial aneurysm by three-dimensional CT angiography. RAA: right atrial aneurysm.

An 11-month-old female child, the offspring of a consanguineous union, was referred to our hospital for investigation of the etiology of her jaundice. There is no family history of liver disease. Physical examination revealed a weight and height below the 3rd percentile and an icteric appearance, with the skin of the entire body covered with excoriated areas due to scratching. Cardiologic examination revealed a 2/6 systolic murmur. Additionally, the liver was palpable 5 cm below the costal margin. Electrocardiography results were normal. The patient, who had signs of right atrial dilatation, underwent echocardiography, which revealed a giant right atrial aneurysm with thin walls, measuring 3.4 × 2.7 cm in size. It was located at the free wall of the right atrium over the tricuspid valve. The large aperture of the aneurysm was connected to the right atrium. It was mildly compressing the right ventricle and tricuspid valve (Figure 1). 3-dimensional CT angiography confirmed the giant aneurysm connected to the right atrium, measuring 4.2 × 2.3 cm in size (Figures 2 and 3). Meanwhile, results of blood analysis for the jaundice were as follows: total bilirubin, 17.1 mg/dl; direct bilirubin, 13.9 mg/dl; alanine aminotransferase, 120 IU/L; aspartate aminotransferase, 82 IU/L; and gamma glutamyl transferase, 20 IU/L. Abdominal ultrasonography yielded normal results except for hepatomegaly. Magnetic resonance cholangiography findings were also normal. There were no butterfly vertebrae on chest radiographs or facial features suggestive of Alagille syndrome. Results of vision and hearing examinations with brainstem-auditory evoked responses were also unremarkable. Tests showed normal renal function and normal blood sugar. Histological examination of liver biopsy specimens suggested progressive familial intrahepatic cholestasis type 3. The patient was started on ursodeoxycholic acid and kept under close observation.

## 3. Discussion

Both giant right atrial aneurysm and progressive familial intrahepatic cholestasis are rare entities. Their comorbidity has not been reported before in the literature. While the etiology of right atrial aneurysm is unclear, progressive familial intrahepatic cholestasis is a disease inherited in an autosomal recessive pattern [2, 5]. The coexistence of both conditions in our patient is interesting. While patients with giant right atrial aneurysms may be asymptomatic, they may also die from early complications of the disease or may be misdiagnosed with other diseases included in the differential diagnosis [3, 4]. Patients with progressive familial intrahepatic cholestasis or right atrial aneurysms should be investigated for signs of other diseases that are asymptomatic or have not developed clinical signs.

## Additional Points

*Learning Objectives*. Both giant right atrial aneurysm and progressive familial intrahepatic cholestasis are rare entities. Their comorbidity has not previously been reported in the literature, and we are therefore reporting the case.
